# The impact of puberty on the onset, frequency, location, and severity of attacks in hereditary angioedema due to C1-inhibitor deficiency: A survey from the Italian Network for Hereditary and Acquired Angioedema (ITACA)

**DOI:** 10.3389/fped.2023.1141073

**Published:** 2023-04-18

**Authors:** Mauro Cancian, Paola Triggianese, Stella Modica, Francesco Arcoleo, Donatella Bignardi, Luisa Brussino, Caterina Colangelo, Ester Di Agosta, Davide Firinu, Maria Domenica Guarino, Francesco Giardino, Marica Giliberti, Vincenzo Montinaro, Riccardo Senter

**Affiliations:** ^1^ UOSD Allergologia, University Hospital of Padua, Padua, Italy; ^2^ Tor Vergata University Hospital, Rome, Italy; ^3^ UOC di Patologia Clinica e Immunologia, AOR Villa Sofia-Cervello, Palermo, Italy; ^4^ IRCCS Ospedale Policlinico San Martino, Genova, Italy; ^5^ University Hospital Ordine Mauriziano di Torino, Turin, Italy; ^6^ Azienda Sanitaria Locale di Pescara, Pescara, Italy; ^7^ Immunoallergology unit, Careggi University Hospital, Florence, Italy; ^8^ Department of Medical Sciences and Public Health, University of Cagliari, Cagliari, Italy; ^9^ Ospedale di Civitanova Marche, Civitanova Marche, Italy; ^10^ Azienda Ospedaliero-Universitaria Policlinico “G.Rodolico-San Marco”, Catania, Italy; ^11^ Azienda Ospedaliero-Universitaria Policlinico di Bari, Bari, Italy; ^12^ Ospedale Regionale Generale F Miulli, Acquaviva delle Fonti, Italy

**Keywords:** C1-inhibitor (C1INH), contact system, rare disease, hereditary angioedema (HAE), pediatric, puberty, ITACA

## Abstract

**Introduction:**

Hereditary angioedema due to C1-inhibitor deficiency is influenced by hormonal factors, with a more severe course of disease in women. Our study aims to deepen the impact of puberty on onset, frequency, location and severity of attacks.

**Methods:**

Retrospective data were collected through a semi-structured questionnaire and shared by 10 Italian reference centers of the Italian Network for Hereditary and Acquired Angioedema (ITACA).

**Results:**

The proportion of symptomatic patients increased significantly after puberty (98.2% vs 83.9%, *p*=0.002 in males; 96.3% vs 68,4%, *p*<0.001 in females); the monthly mean of acute attacks was significantly higher after puberty, and this occurred both in females (median (IQR) = 0.41(2) in the three years before puberty vs 2(2.17) in the three years after, *p*<0.001) and in males (1(1.92) vs 1.25(1.56) respectively, *p*<0.001). The increase was greater in females. No significant differences were detected in attack location before and after puberty.

**Discussion:**

Overall, our study confirms previous reports on a more severe phenotype in the female gender. Puberty predisposes to increased numbers of angioedema attacks, in particular in female patients.

## Introduction

The term angioedema (AE) comes from the union of the two ancient Greek words *αγ*γ*ɛ*ί*ον* (vessel) and *ο*ί*δημα* (edema) to emphasize how, unlike cardiovascular or renal edema that are driven by oncotic pressure and Starling's law, it is a disease that results from specific alterations in endothelial permeability (1). In most cases, especially in the pediatric population, AE is due to histamine release from mast cells and basophils, both when associated with urticaria and when it occurs in isolated manner (2, 3).

However, there are also some endotypes of AE mediated by bradykinin (BK), a vasoactive peptide produced in the circulation from high-molecular-weight kininogen (HMWK) upon activation of the contact system (4, 5). Angiotensin converting enzyme (ACE)-inhibitors (ACEI), which reduces BK catabolism by blocking ACE, the main proteases of BK, are the most frequent cause of bradykininergic AE (6, 7). Other drugs—among which gliptins, neprilysin inhibitors, and angiotensin-II-receptor antagonists—may also be involved in the pathogenesis of BK-mediated, iatrogenic angioedema that seldom affects pediatric population (8).

Infants, children and adolescents, on the other hand, represent an age cohort involved in hereditary angioedema due to C1-inhibitor (C1INH) deficiency (HAE-C1INH), which is also mediated by BK as the terminal effector (9, 10).

The history of HAE-C1INH is a long journey that started in 1882 with the first cases of angioedema in the modern era of medicine reported by Henrick Quincke, whose name later became the eponym for the disease (11). As thoroughly resumed by Reshef et al. (12), in the following decades Sir William Osler described the familiarity of the disease and Crowder the autosomal dominant transmission, but it took a century before the discovery of the pathogenetic roles of C1INH and bradykinin, respectively, by Virginia Donaldson in 1964 and Erich Nussberger in 1998. In the 2000s, six new hereditary variants of hereditary angioedema with normal C1INH have been identified to date since the mutation in the gene for factor XII described by Konrad Bork and Karen E. Binkley (13–15). Hereditary forms of angioedema of unknown origin also exist, but the most common one—although rare with an estimated prevalence of around 1:50,000—still by far remains HAE-C1INH (7, 16–21).

Although the first physiologic action of C1INH to be discovered consists in blocking the activation of the complement cascade, this protease inhibitor belonging to the serpin family exerts many actions on the contact system: in particular, it blocks the enzymatic activity of factor XII and kallikrein, thus reducing the generation of bradykinin from HMWK (22–24). Reduced or absent C1INH activity results, therefore, in increased BK production, predisposing to plasma leakage from the microcirculation (25, 26). When in HAE-C1INH, as well as in some hypertensive patients taking ACEI or in acquired forms of C1INH deficiency—even rarer than HAE (1:10) and not affecting the pediatric population as usually secondary to lymphoproliferative or autoimmune diseases (27, 28)—an additional stimulus of unknown nature locally adds, the plasma leaks from the capillary and elicits angioedema.

There is no itching or wheals, and many districts can be affected: subcutaneous tissue, with painful swelling and functional limitation that can persist several days if untreated; gastrointestinal and urogenital submucosa, with abdominal colic mimicking an acute abdomen and often leading to inappropriate surgery; upper airways, with life-threatening attacks when larynx is involved (29–32). The most common triggers of angioedema—which may be preceded by prodromes as erythema marginatum, irritability, sense of hunger, asthenia, or other signs and symptoms—are trauma, surgery, airway endoscopy, dental procedures, infections, and stressful conditions. In most cases, however, the onset of acute attacks occurs spontaneously and without any presaging sign (33–35).

C1INH deficiency may be due to impaired production of C1INH (HAE-C1INH type 1, 85% of cases) or to the synthesis of a dysfunctional protein (HAE-C1INH type 2) (1, 10). Both C1INH-HAE type 1 and type 2 have decreased C1INH activity. C1INH-HAE type 1 has a low amount of plasma C1INH caused by the lack of production from the mutated allele or production of a protein without function unrecognized in the antigen analysis. C1INH-HAE type 2 has low amount of functional C1INH in plasma and usually increased amounts of abnormal C1INH as this C1INH protein is unable to make complexes with target proteases. The phenotype and pattern of inheritance are the same, but the diagnostic approaches are different: while in HAE-C1INH type 1 it is sufficient to demonstrate an antigenic deficiency of the protein by turbidimetry, nephelometry, radial immunodiffusion, immunoblotting, or other immunologic methods, the diagnosis of HAE-C1INH type 2 requires assessment of functional C1INH activity by chromogenic or ELISAs, which need special attention even at the preanalytical stage (1, 10, 36–39). The C4 plasma concentration measurement, which is cheap, easy to perform, and worldwide available although nonspecific, can be very useful as a screening test because its level is reduced in around 95% of C1INH-deficient patients (19, 38–40). Finally, although HAE-C1INH is a genetic disease, mutational analysis of *Serping1*, the gene on chromosome 11 that encodes for C1INH, is usually not necessary apart from, in rare cases of doubt, prenatal (chorionic villous sampling or amniocentesis) or preimplantation diagnosis of HAE and scientific purposes (1, 10, 39–43). Moreover, gene assessment may be helpful in the first year of life because too early testing in newborns may lead to a false diagnosis of HAE-C1INH (44, 45).

Despite diagnostic advances, including new tools that extend the possibility of diagnosis to reference centers and physicians who eventually lack the proper facilities (46, 47), the diagnostic delay in HAE-C1INH still remains significant as typical of rare diseases (48). In patients with type II HAE-C1INH and in the *de novo* mutations, which represents up to 20% of cases and have no parents affected, the interval between symptom onset and diagnosis has been reported to be even greater (19, 44, 49).

Although the majority of data and studies regarding hereditary angioedema are based on adults, HAE-C1INH is a disease of pediatric interest, as typical of most congenital diseases. The very early occurrence of potential life-threatening attacks, the unpredictable nature of acute episodes, and their negative consequences on normal daily, school and social activities contribute to a significant disease burden and reduced quality of life (QoL) for both young patients and their parents, relatives, and caregivers (50–52). The onset of symptoms is reported to present in most cases in childhood, with median age of the first swelling ranging from 5 to 11 years and mean diagnostic age of diagnosis and diagnostic delay highly variable in different surveys (4, 53–60). However, these retrospective studies involve very limited populations and are so heterogeneous in their methods that they are difficult to compare with each other, and are not focused on the influence of puberty, which is expected to be relevant due to hormonal influences, psychological aspects, and the possible introduction of estroprogestin in the female population.

Our study aims to deepen our understanding of puberty on the onset, frequency, location, and severity of attacks in hereditary angioedema due to C1-inhibitor deficiency utilizing data shared by the Italian Network for Hereditary and Acquired Angioedema (ITACA, www.angioedemaitaca.org), which brings together Italian reference centers and uses an electronic disease registry where patients can directly enter data on their clinical course.

## Patients and methods

This multicenter, retrospective observational study was performed on HAE patients from 10 ITACA centers throughout Italy, thus minimizing the potential bias caused by regional differences.

Inclusion criteria were age between 10 and 40 years, puberty already occurred, and enrollment in the ITACA Registry, approved by the ethics committee of the coordinating center (Comitato etico Milano area 1) on 5 May 2017. The patients were interviewed from 1 to 30 August 2022 by specialized personnel who administered a semistructured questionnaire (Supplementary Table S1) addressing patient characteristics and the effect of puberty on attack location, severity and frequency, related treatments, and overall burden of disease. Age of onset of puberty was defined as the age at menarche for females and the age at semenarche for males. In order to estimate attacks frequency, the patients were asked to report a monthly mean of attacks in the year and in the 3 years before and after puberty. All patients had previously provided written consent to be included in the registry and reiterated verbal consent at the time of the interview. For patients younger than 18 years, interviews were performed in the presence and with the consent of parents.

### Statistical analysis

Continuous variables are shown as mean and SD or median and interquartile range (IQR) depending on their distribution. Shapiro–Wilk test was used to check if the variables followed a normal distribution. Categorical variables are shown as number and percentage. Student's *t*-test or Mann–Whitney test were used for comparisons between continuous variables, depending on their distribution; Chi-square test was used for comparisons between categorical variables (Fisher's exact test when the number in any cell was smaller than 5). Binomial logistic regression was used to investigate the ability of a set of predictor variables to predict the outcome. All the analyses performed are detailed in Supplementary Table S2. Statistical analysis was performed with Jamovi [The jamovi project (2021). *jamovi* Version 1.6]. *p* ≤ 0.05 was considered statistically significant.

## Results

### Population characteristics

Out of 147 eligible patients referring to the centers involved in the study, 118 (60 F, 58 M; HAE-C1INH type 1 = 113, type 2 = 5) were enrolled. As in Table 1, demographic and clinical characteristics were similar between males and females, but the age of puberty was lower in the female gender (median: 12 years in females vs. 13 years in males, *p* < 0.001); median age at the interview was 30 years (IQR = 13,75) in males and 29.5 years (IQR = 11,25) in females (*p* = 0.779); median age at first attack was 6 years (IQR = 6,5) in males and 8 years (IQR = 10) in females.

**Table 1:** Demographic and clinical characteristics of patients.

	Male	Female	All	*p* value
Subjects, number (%)	58 (49)	60 (51)	118 (100)	
Current age (years), median (IQR); *n* = 118	30 (13.75)	29.5 (11.25)	30 (13)	0.779
Type of angioedema, number (% within column); *n* = 118				0.677
HAE-C1INH type I	55 (94.8)	58 (96.7)	113 (95.8)	
HAE-C1INH type II	3 (5.2)	2 (3.3)	5 (4.2)	
Angioedema age of onset (years), median (IQR); *n* = 114	6 (6.5)	8 (10)	7 (8.75)	0.235
Asymptomatic, number (%)	2 (3.4)	2 (3.3)	4 (3.4)	0.976
Age at diagnosis (years), median (IQR); *n* = 118	9 (12.5)	8 (15)	8.5 (13)	0.676
Delay between first symptom and diagnosis (months), median (IQR); *n* = 94	18 (81)	24 (83)	24 (81)	0.832
Plasma C1-inhibitor antigen at diagnosis (mg/dL), median (IQR); *n* = 72^a^	5.8 (4.07)	6.78 (4.27)	6.4 (4.34)	0.340
Plasma C1-inhibitor function at diagnosis (%), mean (SD); *n* = 77	22.85 (14.65)	28.52 (16.45)	25.50 (15.67)	0.101
Plasma C4 component at diagnosis (mg/dL), mean (SD); *n* = 77	7.08 (3.28)	6.116 (2.67)	6.61 (3.02)	0.219
Familiarity, number (% within column); *n* = 117				0.871
*De novo* mutations	12 (20.7)	10 (16.9)	22 (18.8)	
Father affected	24 (41.4)	(44.1)	50 (42.7)	
Mother affected	22 (37.9)	23 (39)	45 (38.5)	
Status of proband, number (% within column); *n* = 118	16 (27.6)	17 (28.3)	33 (28)	0.928
Number of siblings, median (IQR); *n* = 104	1 (1)	1 (1)	1 (1)	0.216
Number of affected siblings, median (IQR); *n* = 107	1 (1)	0 (1)	0 (1)	0.041
Puberty age of onset (years), median (IQR); *n* = 117	13 (2)	12 (1.5)	13 (1)	<0.001
First HAE-C1INH treatment used, number (% within column); *n* = 118				0.958
Plasma-derived C1INH	32 (55.2)	34 (56.7)	66 (55.9)	
Tranexamic acid	13 (22.4)	14 (23.3)	27 (22.9)	
Icatibant	*6* (*10.3)*	3 (5)	9 (7.6)	
Plasma-derived C1INH + icatibant	2 (3.4)	2 (3.3)	4 (3.4)	
Plasma-derived C1INH + tranexamic acid	1 (1.7)	1 (1.7)	1 (1.7)	
Fresh frozen plasma	0	1 (1.7)	1 (0.8)	
None	4 (6.9)	5 (8.3)	9 (7.6)	
Beginning of HAE treatment before puberty, number (% within column); *n* = 107	27 (50)	25 (47.2)	52 (48.6)	0.770
**Comorbidities (number)**				
Breast cancer	0	1	1	
Autoimmune conditions^b^	1	5	6	
Congenital diseases^c^	3	1	4	
Hormonal disorders^d^	1	6	7	
**Concomitant medications (number)**				
Non-hormonal chemotherapy	0	1	1	
Levothyroxine	0	3	3	
DIMARDs^e^	1	2	3	
Hormones^f^	1	6	7	

IQR, interquartile range; HAE-C1INH, hereditary angioedema due to C1-inhibitor deficiency; INH, inhibitor; HAE, hereditary angioedema.

^a^ Four subjects with functional deficit were not considered.

^b^ Hashimoto thyroiditis, unspecified arthritis, diabetes insipidus, Sjogren syndrome, and lupus erythematosus.

^c^ Arrhythmia, neurological, and hematological.

^d^ Endometriosis, hypogonadism, and polycystic ovary syndrome.

^e^ Disease modifying antirheumatic drugs: hydroxichlorquine and sulphasalazyne.

^f^ Etinilestradiol/drospirenone, unspecified progestin, testosterone, and desmopressin.

### The impact of puberty on HAE: overall clinical picture

The proportion of symptomatic patients increased significantly after puberty in both males and females (97.3% vs. 76.1%, *p* < 0.001 in the whole cohort; 98.2% vs. 83.9%, *p* = 0.002 in males; 96.3% vs. 68.4% *p* < 0.001 in females) (Tables 2, 3). In the 24 patients who became symptomatic after puberty, the first attack occurred after a mean of 5.14 years and a median of 3.5 years.

**Table 2:** Angioedema attacks and treatments before puberty among male and female subjects.

	Male	Female	All	*p* value
Monthly mean number of attacks in the year before puberty, median (IQR); *n* = 113	1 (1.92)	0.5 (2)	1 (2)	0.113
Monthly mean number of attacks in the 3 years before puberty, median (IQR); *n* = 113	1 (1.92)	0.41 (1)	1 (2)	0.048
Main site of attacks, number (% within column); *n* = 113				0.057
Cutaneous and abdominal	24 (42.9)	10 (17.5)	34 (30.1)	
Cutaneous	10 (17.9)	16 (28.1)	26 (23)	
Abdominal	9 (16.1)	8 (14)	17 (15)	
Upper airways	4 (7.2)	5 (8.9)	9 (8)	
No attacks	9 (16.1)	18 (31.6)	27 (23.9)	
Most common on-demand treatment, number (% within column); *n* = 102				0.349
Plasma-derived C1INH	18 (34)	10 (20.4)	28 (27.5)	
Tranexamic acid	8 (15.1)	7 (14.3)	15 (14.7)	
Plasma-derived C1INH and tranexamic acid	2 (3.8)	4 (8.2)	6 (5.9)	
Icatibant	1 (1.9)	0 (0)	1 (1)	
Plasma-derived C1INH and icatibant	1 (1.9)	0 (0)	1 (1)	
None	23 (43.4)	28 (57.1)	51 (50)	
Any long-term prophylaxis before puberty, number (% within column); *n* = 118	3 (5.2)	4 (6.7)	7 (5.9)	0.731
Long-term prophylaxis before puberty, number (% within column); *n* = 118				0.562
Tranexamic acid	2 (3.4)	2 (3.3)	4 (3.4)	
Plasma-derived C1INH and tranexamic acid	0 (0)	1 (1.7)	1 (0.8)	
Danazol	1 (1.7)	0 (0)	1 (0.8)	
Unspecified	0 (0)	1 (1.7)	1 (0.8)	
None	55 (94.8)	56 (93.3)	111 (94.1)	
Hospital accesses before puberty, median (IQR); *n* = 90	0 (1)	0 (0)	0 (1)	0.305
At least one hospital access before puberty, number (% within column); *n* = 118	25 (43.1)	18 (30)	43 (36.4)	0.139

IQR, interquartile range; C1INH, C1-inhibitor.

**Table 3:** Angioedema attacks and treatments after puberty among male and female subjects.

	Male	Female	All	*p* value
Monthly mean number of attacks in the year after puberty, median (IQR); *n* = 113	1 (2.65)	1 (2.46)	1 (2.75)	0.764
Monthly mean number of attacks in the 3 years after puberty, median (IQR); *n* = 111	1.25 (1.56)	2 (2.17)	2 (2.42)	0.327
Main site of attacks, number (% within column); *n* = 110				0.496
Cutaneous and abdominal	28 (50)	20 (37)	48 (43.6)	
Cutaneous	8 (14.3)	13 (24.1)	21 (19.1)	
Abdominal	12 (21.4)	14 (25.9)	26 (23.6)	
Upper airways	7 (12.5)	5 (9.3)	12 (10.9)	
No attacks	1 (1.8)	2 (3.7)	3 (2.7)	
Most common on-demand treatment after puberty, number (% within column); *n* = 106				0.947
Plasma-derived C1INH	29 (53.7)	28 (53.8)	57 (53.8)	
Tranexamic acid	1 (1.9)	2 (3.8)	3 (2.8)	
Plasma-derived C1INH and tranexamic acid	2 (3.7)	2 (3.8)	4 (3.8)	
Icatibant	11 (20.4)	8 (15.4)	19 (17.9)	
Plasma-derived C1INH and icatibant	6 (11.1)	7 (13.5)	13 (12.3)	
Plasma-derived C1INH, icatibant, and tranexamic acid	1 (1.9)	0 (0)	1 (0.9)	
None	4 (7.4)	5 (9.6)	9 (8.5)	
Long-term prophylaxis in the 3 years after puberty, number (% within column); *n* = 118	5 (8.6)	11 (18.3)	16 (13.6)	0.123
Long-term prophylaxis in the 3 years after puberty, number (% within column); *n* = 118				0.099
Tranexamic acid	0 (0)	5 (8.3)	5 (4.2)	
Danazol	4 (6.9)	3 (5)	7 (5.9)	
Lanadelumab	1 (1.7)	0 (0)	1 (0.8)	
Unspecified	0 (0)	1 (1.7)	1 (0.8)	
None	53 (91.4)	49 (81.7)	102 (86.4)	
Hospital accesses in the year after puberty, median (IQR); *n* = 102	0 (0.50)	0 (0)	0 (0)	0.718
Hospital accesses in the 3 years after puberty, median (IQR); *n* = 95	0 (0)	0 (0)	0 (1)	0.560

IQR, interquartile range; C1INH, C1-inhibitor.

**Figure 1:**
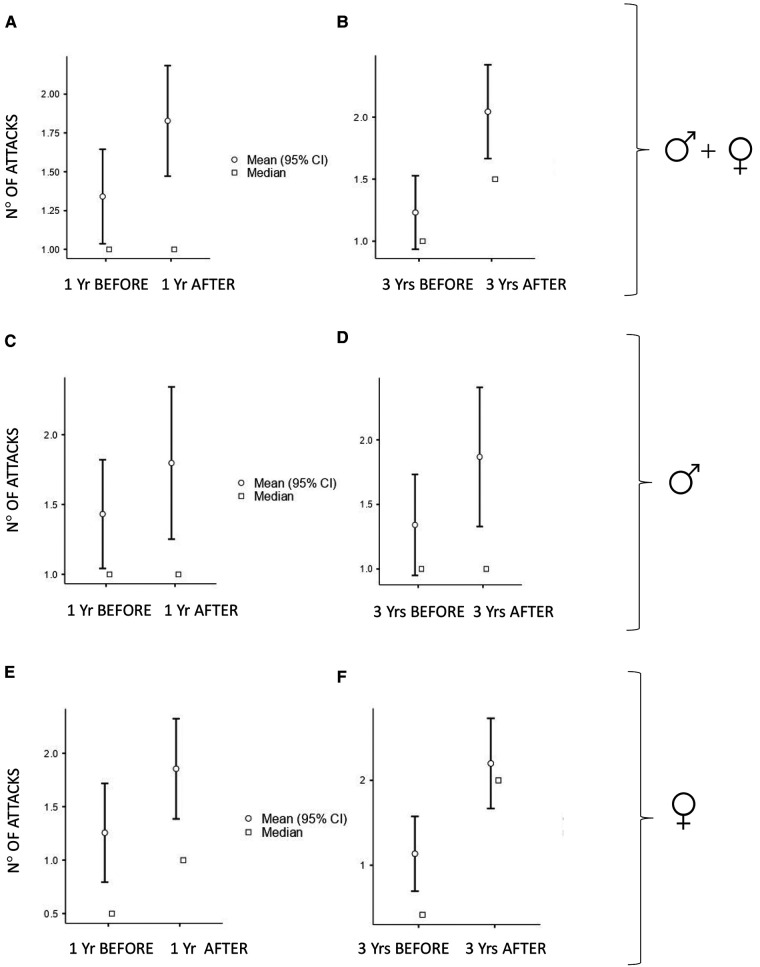
Number of monthly mean of attacks 1 year (Yr) before vs. 1 year after puberty and 3 years (Yrs) before vs. 3 years after puberty: In all the subjects: (**A**) median (IQR) = 1 (2) vs. 1 (2.75), *p* < 0.001 and (**B**) median (IQR) = 1 (2) vs. 2 (2.42), *p* < 0.001. In males: (**C**) median (IQR) = 1 (1.92) vs. 1 (2.65), *p* = 0.035 and (**D**) median (IQR) = 1 (1.92) vs. 1.25 (1.56), *p* < 0.001. In females: (**E**) median (IQR) = 0.5 (2) vs. 1 (2.46), *p* = 0.004 and (**F**) median (IQR) = 0.41 (2) vs. 2 (2.17), *p* < 0.001.

The numbers of acute attacks were significantly higher after puberty when comparing both 1-year and 3-year periods pre/after menarche/semenarche (*p* < 0.001 and *p* < 0.001 in the whole cohort; *p* = 0.035 and *p* < 0.001 in males; *p* = 0.004 and *p* < 0.001 in females) (Figure 1).

**Figure 2:**
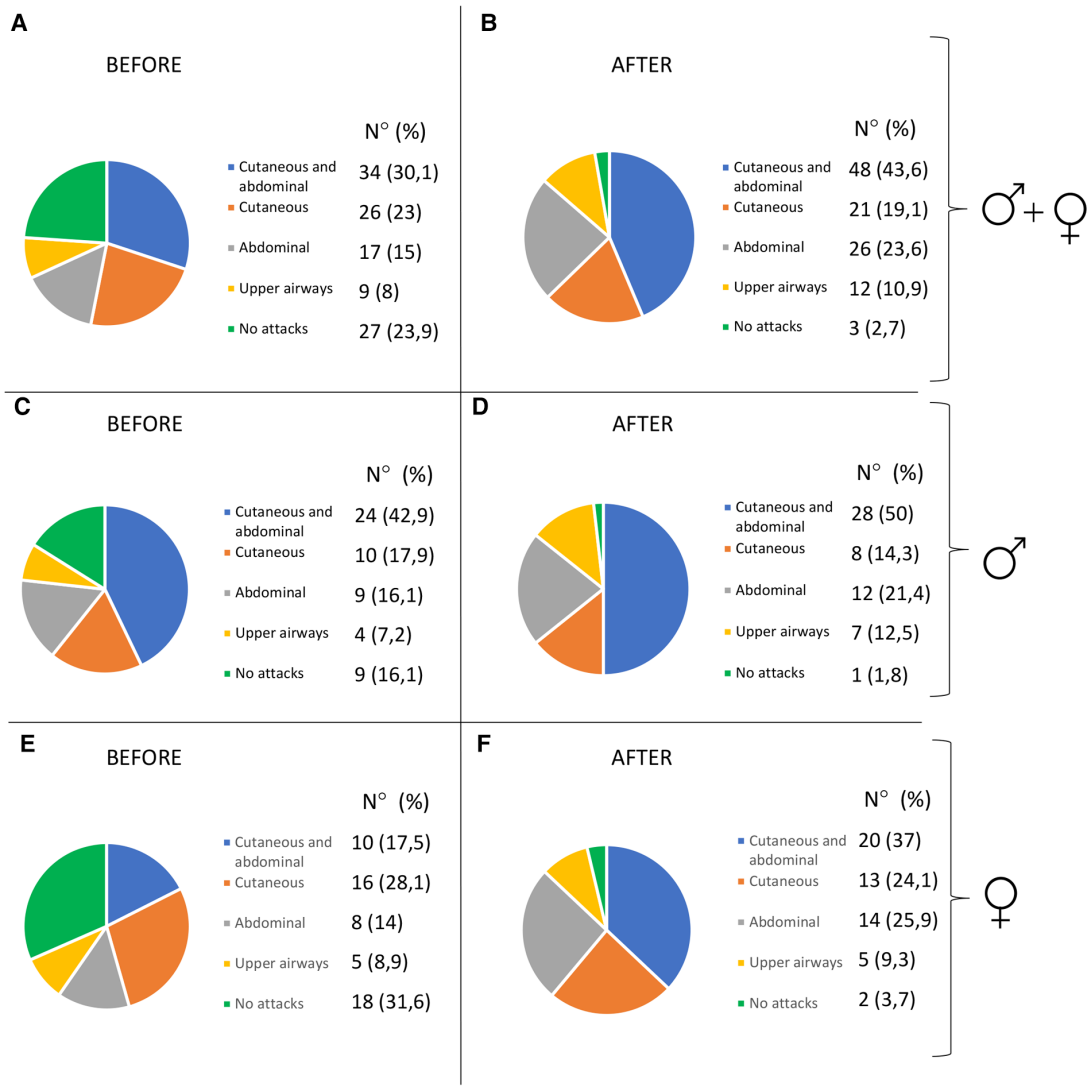
Pattern of attacks before and after puberty in all the patients (**A,B**), in males (**C,D**), and in females (**E,F**).

Characteristics of HAE attacks and related treatments before puberty are reported in Table 2, and after puberty in Table 3. No significant differences were detected in the attack location before and after puberty (Figure 2).

### The impact of puberty: gender differences

**Figure 3:**
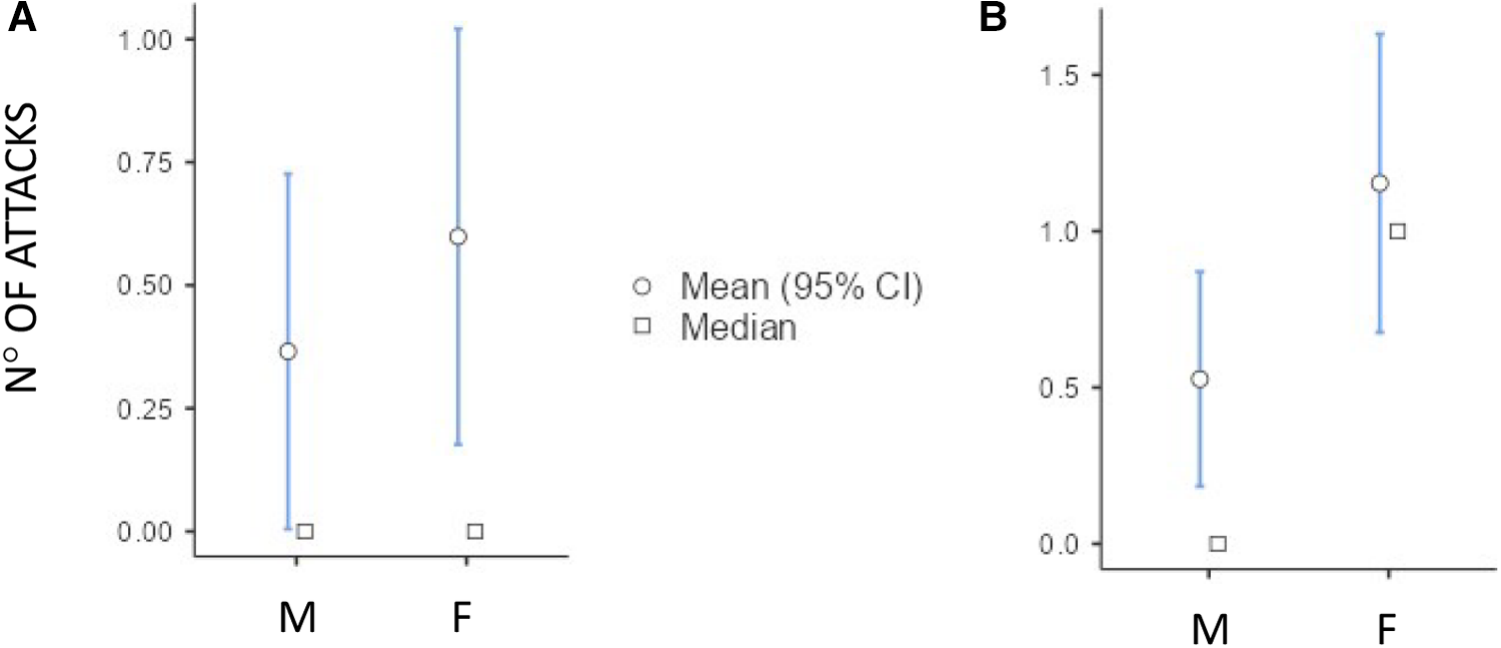
Increase of the number of monthly mean of attacks in male (M) and female (F) between 1 year before vs. 1 year after puberty [(**A**) median (IQR) = 0 (0.5) vs. 0 (1), *p* = 0.174] and 3 years before vs. 3 years after puberty [(**B**) median (IQR) = 0 (1) vs. 1 (2) *p* = 0.02].

**Figure 4:**
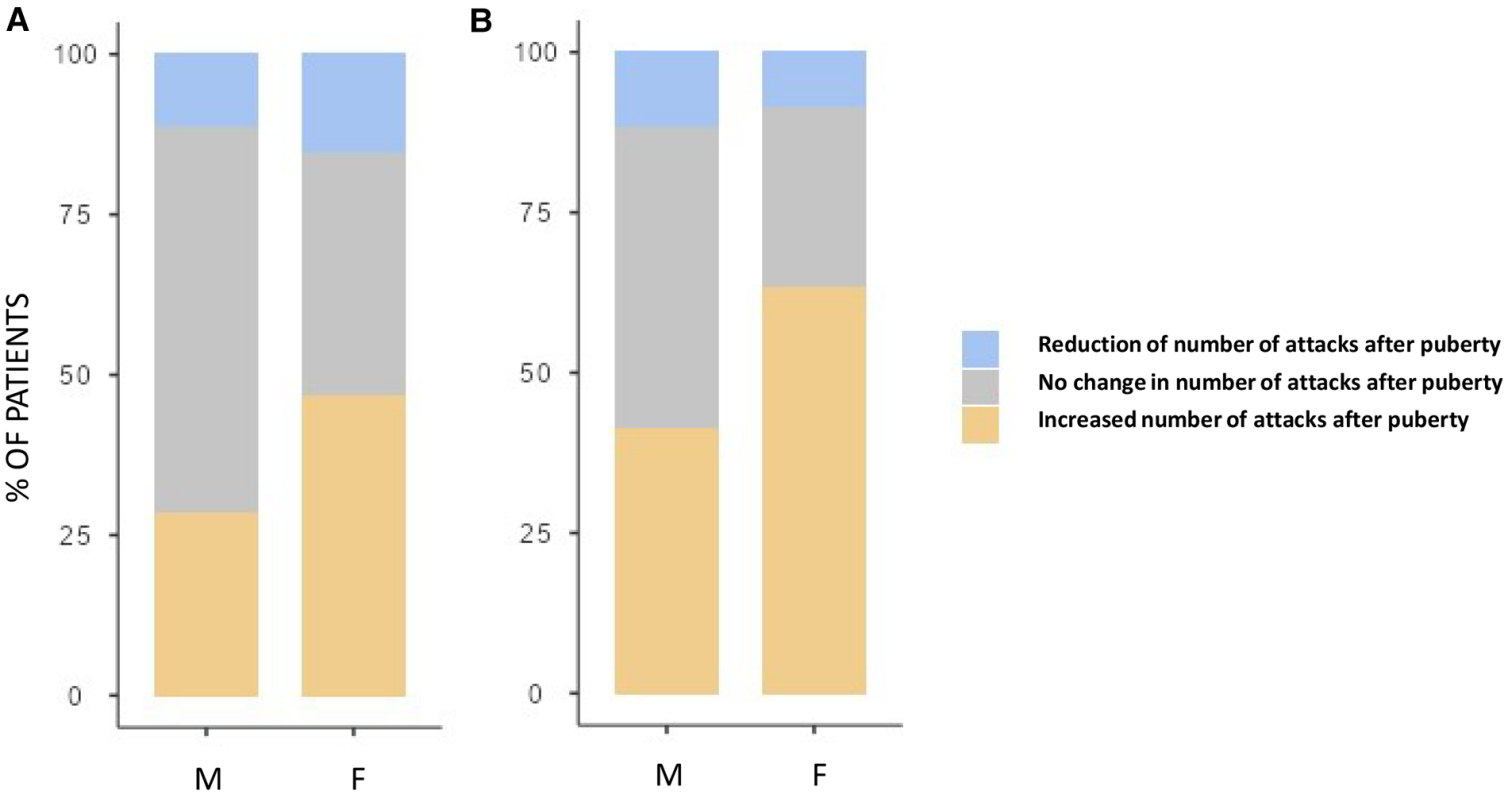
Proportion of subject reporting a reduction (blue), an increase (orange), or no change (gray) in the monthly mean number of attacks after puberty in male (M) and female (F) between 1 year before vs. 1 year after puberty (**A**) and 3 years before vs. 3 years after puberty (**B**) Significantly more females reported an increase of attacks in both periods: 28.3% vs. 46.6%, *p* = 0.048 and 41.2% vs. 63.2%, *p* = 0.022, respectively.

Although both the genders showed an increase in the number of HAE attacks after puberty, this occurred more in females than in males as detailed in Figure 3 (differences in monthly mean of acute episodes of angioedema pre/post puberty) and Figure 4 (percentage of males and females in which the number of attacks varies pre/post puberty). Specifically, the median increase of monthly mean of attacks between 3 years before and 3 years after puberty was 1 (IQR = 2) in females and 0 (IQR = 1) in males (*p* = 0.02); the increase was present, even if not significant, also considering 1 year before and after puberty. Moreover, significantly more females reported an increase of attacks in both periods: in the 3 years after puberty attacks were more frequent in 63.2% of the females and in 41.2% of the males (*p* = 0.022).

Nevertheless, no substantial differences were detected between males and females with regard to their subjective perception of illness before and after puberty (Table 4).

**Table 4:** Overall impact of puberty among male and female subjects.

	Male	Female	All	*p* value
Difference in the number of monthly mean of attacks between 1 year before vs. 1 year after puberty, median (IQR) *n* = 109	0 (0.5)	0 (1)	0 (1)	0.174
Difference in the number of monthly mean of attacks between 3 years before vs. 3 years after puberty, median (IQR) *n* = 106	0 (1)	1 (2)	0.125 (1)	0.020
Patients in which the number of attacks varies after 1 year from puberty, number (% within column); *n* = 111				0.059 (0.048 when considering increase vs. no change + reduction)
Reduction	6 (11.3)	9 (15.5)	15 (13.5)	
No change	32 (60.4)	22 (37.9)	54 (48.6)	
Increase	15 (28.3)	27 (46.6)	42 (37.8)	
Patients in which the number of attacks varies after 3 years from puberty, number (% within column); *n* = 108				0.070 (0.022 when considering increase vs. no change + reduction)
Reduction	6 (11.8)	5 (8.8)	11 (10.2)	
No change	24 (47.1)	16 (28.1)	40 (37)	
Increase	21 (41.2)	36 (63.2)	57 (52.8)	
Subjective feelings of symptoms worsening after puberty, number (% within column); *n* = 114	23 (41.1)	32 (55.2)	55 (48.2)	0.132
Symptoms worsening permanently, number (% within column); *n* = 114	18 (32.1)	23 (39.7)	41 (36)	0.403
Type of Symptoms worsening, number (% within column); *n* = 55				
Frequency of attacks	8 (34.8)	10 (31.3)	18 (32.7)	0.456
Increased duration/intensity of attacks	1 (4.3)	0 (0)	1 (1.8)	
Both	14 (60.9)	22 (68.8)	36 (65.5)	

### The impact of puberty: predictive factors

In regression analysis, female gender predicts the worsening of symptoms for both 1-year and 3-year periods after puberty (OR 2.2, Std Err 1.47–3.3, *p* = 0.049 and OR 2.44, Std Err 1.65–3.63, *p* = 0.024, respectively). This is true for the 3-year period also when considering age of first menstruation as covariate [OR 2.43, Std Err 1.61–3.68, *p* = 0.031].

## Discussion

Our retrospective survey in a large group of patients with HAE-C1INH from the ITACA cohort addresses the impact of puberty on the course of the disease.

Puberty is a dynamic, physical, and psychological growth process lasting several years and not a single event at a specific age (59). However, we decided to identify a significant feature of puberty easy to be recognized and remembered in order to assess the impact of puberty on patient symptoms in our survey. Thus, we chose the onset of menstruation and semenarche as the key indicator in pubertal development for their biological, psychological, and relational relevance.

The mean age indicated for these two events by our study population, 12 and 13 years for males and females (Table 1), was not different from data reported in the overall population (61–63). This finding is consistent with the empirical evidence from clinical practice of normal sexual development of children affected by HAE-C1INH.

The frequency of HAE attacks before puberty was similar between males and females, with numbers of acute episodes slightly higher in males in the prepubertal 3-year period (median of monthly mean: M = 1.0; F = 0.41; *p* = 0.048; Table 2). However, female gender predicts a worsening of symptoms in the 1-year and 3-year period after puberty compared to the same pre-puberty time intervals (*p* = 0.049 and 0.024, respectively). Symptom worsening after puberty occurred to a greater extent in female subjects than in males (Figures 3, 4). On the contrary, there was no significant variation in the location of the attacks, which were reported mostly as abdominal and subcutaneous both before and after puberty by both the genders, similar to what is described for HAE-C1INH patients of any age and sex (10) (Figure 2).

Previous data suggested that adults present a higher frequency of acute episodes of angioedema than the pediatric population (64–66), although a thorough comparison between pediatric and adult patients is still lacking. Among the several biological factors involved in the process of growth that could theoretically contribute to this increase, hormonal factors certainly play a role (67–70). In fact, estrogen hormones promote a pattern of protein expression, which ultimately increases the activity of the contact system (71, 72) and female patients tend to present a more severe disease (1). In the present manuscript, we show that puberty is a watershed moment of life when the disease starts to get worse for women. Moreover, in our cohort, puberty seemed to represent a turning point also for male subjects: although on semenarche an androgen-dominated pattern of hormones is secreted (73), we might speculate that the bioactive metabolite of testosterone estradiol can act on the contact system and worsen symptoms in the male gender.

Finally, we were not able to demonstrate a parallel worsening of QoL in the period after menarche and semenarche compared to the prepubertal situation. This is because it is well recognized that the frequency of attacks is not the only factor affecting patient QoL, as the severity of single HAE episodes and the unpredictability of their onset play a crucial role in the global burden of disease (52). Moreover, the study design did not allow us to use specific tools to assess QoL variations.

Further limitations of the study derive from the retrospective approach and the approximate quantification of the number of attacks not included in the register, active since 2017, especially in patients who had been through puberty for over a decade. Several biases could influence the accuracy of attack frequency, when retrospectively reported. Among others, subjects may had been influenced by the interviewers, and attacks occurred in young age might be more easily forgotten.

Moreover, treatment strategies were highly variable, also because of the many new drugs developed in recent years. Thus, although we tried to limit these biases by including in the study only subjects under 40 years of age, we were well aware that a comparison of on-demand treatment before and after puberty was unreliable and reported only the descriptive statistics. Nevertheless, the prevalence of patients on long-term prophylaxis for HAE-C1INH had raised from 5.9% before puberty to 13.6% in the 3 years after menarche or semenarche (Tables 2, 3), thus strengthening the evidence of symptom worsening after puberty.

Overall, our study confirms previous reports on a more severe phenotype in the female gender and provides new insights on the impact of puberty on a large cohort of HAE patients from the ITACA network. Results show that puberty predisposes to increased numbers of angioedema attacks, in particular in female patients. Retrospective data collection should be confirmed in prospective assessments, which are challenging in the field of rare diseases but may be favored by patient adherence to disease registries.

## Data Availability

The raw data supporting the conclusions of this article will be made available by the authors, without undue reservation.

## References

[1] Zuraw BL Christiansen SC. HAE pathophysiology and underlying mechanisms. Clin Rev Allergy Immunol. (2016) 51:216–29. 10.1007/s12016-016-8561-8 27459852

[2] Pier J Bingemann TA. Urticaria, angioedema, and anaphylaxis. Pediatr Rev. (2020) 41:283–92. 10.1542/pir.2019-0056 32482691

[3] Pattanaik D Lieberman JA. Pediatric angioedema. Curr Allergy Asthma Rep. (2017) 17:60. 10.1007/s11882-017-0729-7 28791569

[4] Long AT Kenne E Jung R Fuchs TA Renné T. Contact system revised: an interface between inflammation, coagulation, and innate immunity. J Thromb Haemost. (2016) 14:427–37. 10.1111/jth.13235 26707513

[5] Hofman Z de Maat S Hack CE Maas C. Bradykinin: inflammatory product of the coagulation system. Clin Rev Allergy Immunol. (2016) 51:152–61. 10.1007/s12016-016-8540-0 27122021 PMC5025506

[6] Montinaro V Cicardi M. ACE inhibitor-mediated angioedema. Int Immunopharmacol. (2020) 78:106081. 10.1016/j.intimp.2019.106081 31835086

[7] Aygören-Pürsün E Magerl M Maetzel A Maurer M. Epidemiology of bradykinin-mediated angioedema: a systematic investigation of epidemiological studies. Orphanet J Rare Dis. (2018) 13:73. 10.1186/s13023-018-0815-5 29728119 PMC5935942

[8] Stone C Brown NJ. Angiotensin-converting enzyme inhibitor and other drug-associated angioedema. Immunol Allergy Clin N Am. (2017) 37:483–95. 10.1016/j.iac.2017.04.006 28687104

[9] Cicardi M Zuraw BL. Angioedema due to bradykinin dysregulation. J Allergy Clin Immunol Pract. (2018) 6:1132–41. 10.1016/j.jaip.2018.04.022 30033914

[10] Busse PJ Christiansen SC. Hereditary angioedema. N Engl J Med. (2020) 382:1136–48. 10.1056/NEJMra1808012 32187470

[11] Quincke H. Uber akutes umschriebenes Hautoedem. Mschr Prakt Dermatol. (1882) 1:160–9.

[12] Reshef A Kidon M Leibovich I. The Story of Angioedema: from Quincke to Bradykinin. Clin Rev Allerg Immunol. (2016) 51:121–39. 10.1007/s12016-016-8553-8 27287037

[13] Binkley KE Davis A. Clinical, biochemical, and genetic characterization of a novel estrogen-dependent inherited form of angioedema. J Allergy Clin Immunol. (2000) 106:546–50. 10.1067/mai.2000.108106 10984376

[14] Bork K Barnstedt SE Koch P Traupe H. Hereditary angioedema with normal C1-inhibitor activity in women. Lancet. (2000) 356:213–7. 10.1016/S0140-6736(00)02483-1 10963200

[15] Bork K Wulff K Möhl BS Steinmüller-Magin L Witzke G Hardt J Novel hereditary angioedema linked with a heparan sulfate 3-O-sulfotransferase 6 gene mutation. J Allergy Clin Immunol. (2021) 148:1041–8. 10.1016/j.jaci.2021.01.011 33508266

[16] Stray-Pedersen A Abrahamsen TG Frøland SS. Primary immunodeficiency diseases in Norway. J Clin Immunol. (2000) 20:477–85. 10.1023/A:1026416017763 11202238

[17] Roche O Blanch A Caballero T Sastre N Callejo D López-Trascasa M. Hereditary angioedema due to C1 inhibitor deficiency: patient registry and approach to the prevalence in Spain. Ann Allergy Asthma Immunol*.* (2005) 94:498–503. 10.1016/S1081-1206(10)61121-0 15875532

[18] Bygum A. Hereditary angio-oedema in Denmark: a nationwide survey. Br J Dermatol*.* (2009) 161:1153–8. 10.1111/j.1365-2133.2009.09366.x 19709101

[19] Zanichelli A Arcoleo F Barca MP Borrelli P Bova M Cancian M A nationwide survey of hereditary angioedema due to C1 inhibitor deficiency in Italky. Orphanet J Rare Dis. (2015) 6(10):11. 10.1186/s13023-015-0233-x PMC4333895 25758562

[20] Schöffl C Wiednig M Koch L Blagojevic D Duschet P Hawranek T Hereditary angioedema in Austria: prevalence and regional peculiarities. Dtsch Dermatol Ges. (2019) 17:416–23. 10.1111/ddg.13815 PMC6850089 30883006

[21] Lumry WR Settipane RA. Hereditary angioedema: epidemiology and burden of disease. Allergy Asthma Proc. (2020) 41(Suppl 1):S08–13. 10.2500/aap.2020.41.200050 33109318

[22] Davis AE Mejia P Lu F. Biological activities of C1 inhibitor. Mol Immunol*.* (2008) 45: 4057–63. 10.1016/j.molimm.2008.06.028 18674818 PMC2626406

[23] Schoenfeld AK Lahrsen E Alban S. Regulation of complement and contact system activation via C1 inhibitor potentiation and factor XIIa activity modulation by sulfated glycans—structure-activity relationships. PLoS One. (2016) 11:e0165493. 10.1371/journal.pone.0165493 27783665 PMC5082678

[24] Karknaukhova E. C1-inhibitor: structure, functional diversity and therapeutic development. Curr Med Chem. (2022) 29:467–88. 10.2174/0929867328666210804085636 34348603

[25] Davis AE. The pathopisiology of hereditary angioedema. Clin Immunol. (2005) 114:3–9. 10.1016/j.clim.2004.05.007 15596403

[26] Cugno M Zanichelli A Foieni F Caccia S Cicardi M. C1-inhibitor deficiency and angioedema: molecular mechanisms and clinical progress. Trends Mol Med*.* (2009) 15:69–78. 10.1016/j.molmed.2008.12.001 19162547

[27] Cugno M Castelli R Cicardi M. Angioedema due to acquired C1-inhibitor deficiency: a bridging condition between autoimmunity and lymphoproliferation. Autoimmun Rev. (2008) 8:156–9. 10.1016/j.aurev.2008.05.003 19014872

[28] Cicardi M Zanichelli A. Acquired angioedema. Allergy Asthma Clin Immunol. (2010) 6:14. 10.1186/1710-1492-6-14 20667117 PMC2925362

[29] Zuraw BL. Clinical practice. Hereditary angioedema. N Engl J Med. (2008) 359:1027–36. 10.1056/NEJMcp0803977 18768946

[30] Rubinstein E Stolz LE Sheffer AL Stevens C Bousvaros A. Abdominal attacks and treatment in hereditary angioedema with C1-inhibitor deficiency. BMC Gastroenterol. (2014) 14:71. 10.1186/1471-230X-14-71 24712435 PMC4101849

[31] Bork K Siedlecki K Bosch S Schopf RE Kreuz W. Asphyxiation by laryngeal edema in patients with hereditary angioedema. Mayo Clin Proc. (2000) 75:349–54. 10.4065/75.4.349 10761488

[32] Bork K. Recurrent angioedema and the threat of asphyxation. Dtsch Arztebl Int*.* (2010) 107: 408–14. 10.3238/arztebl.2010.0408 20589206 PMC2893523

[33] Craig T. Triggers and short-term prophylaxis in patients with hereditary angioedema. Allergy Asthma Proc. (2020) 41(Suppl 1):S30–4. 10.2500/aap.2020.41.200058 33109323

[34] Kemp JG Craig TJ. Variability of prodromal signs and symptoms associated with hereditary angioedema attacks: a literature review. Allergy Asthma Proc. (2009) 30:493–9. 10.2500/aap.2009.30.3278 19843403

[35] Leibovich-Nassi I Golander H Somech R Har-Even D Reshef A. New instruments for the evaluation of prodromes and attacks of hereditary angioedema (HAE-EPA). Clin Rev Alklergy Immunol. (2021) 61:29–39. 10.1007/s12016-021-08843-8 33538950

[36] Gompels MM Lock RJ Morgan JE Osborne J Brown A Virgo PF. A multicentre evaluation of the diagnostic efficiency of serological investigations for C1 inhibitor deficiency. J Clin Pathol*.* (2002) 55: 145–7. 10.1136/jcp.55.2.145 11865013 PMC1769585

[37] Wagenaar-Bos IG Drouet C Aygören-Pursun E Bork K Bucher C Bygum A Functional C1-inhibitor diagnostics in hereditary angioedema: assay evaluation and recommendations. J Immunol Methods*.* (2008) 338: 14–20. 10.1016/j.jim.2008.06.004 18655790

[38] Porebski G Kwitniewski M Reshef A. Biomarkers in hereditary angioedema. Clin Rev Allergy Immunol. (2021) 60:404–15. 10.1007/s12016-021-08845-6 33560480 PMC8272698

[39] Maurer M Magerl M Betschel S Aberer W Ansotegui IJ Aygören-Pürsün E The international WAO/EAACI guideline for the management of hereditary angioedema—the 2021 revision and update. Allergy. (2022) 77:1961–90. 10.1111/all.15214 35006617

[40] Cancian M. Diagnostic and therapeutic management of hereditary angioedema due to C1-inhibitor deficiency: the Italian experience. Curr Opin Allergy Clin Immunol. (2015) 15: 383–91. 10.1097/ACI.0000000000000186 26106828

[41] Loules G Zamanakou M Parsopoulou F Vatsiou S Psarros F Csuka D Targeted next-generation sequencing for the molecular diagnosis of hereditary angioedema due to C1-inhibitor deficiency. Gene. (2018) 667:76–82. 10.1016/j.gene.2018.05.029 29753808

[42] Germenis AE Margaglione M Pesquero JB Farkas H Cichon S Csuka D International consensus on the use of genetics in the management of hereditary angioedema. J Allergy Clin Immunol Pract. (2020) 8:901–11. 10.1016/j.jaip.2019.10.004 31669336

[43] Drouet C López-Lera A Ghannam A López-Trascasa M Cichon S Ponard D *SERPING1* variants and C1-INH biological function: a close relationship with C1-INH-HAE. Front Allergy. (2022) 3:835503. 10.3389/falgy.2022.835503 35958943 PMC9361472

[44] Farkas H Martinez-Saguer I Bork K Bowen T Craig T Frank M International consensus on the diagnosis and management of pediatric patients with hereditary angioedema with C1 inhibitor deficiency. Allergy. (2017) 72:300–13. 10.1111/all.13001 27503784 PMC5248622

[45] Pedrosa M Phillips-Angels E López -Lera A López-Trascasa M Caballero T. Complement study versus *CINH* gene testing for the diagnosis of type I hereditary angioedema in children. J Clin Immunol. (2016) 36:16–8. 10.1007/s10875-015-0222-9 26661330

[46] Lai Y Zhang G Inhaber N Bernstein JA Cwik M Zhou Z A robust multiplexed assay to quantify C1-inhibitor, C1q, and C4 proteins for in vitro diagnosis of hereditary angioedema from dried blood spot. J Pharm Biomed Anal. (2021) 195:113844. 10.1016/j.jpba.2020.113844 33388640

[47] Förster TM Magerl M Maurer M Zülbahar S Zielke S Inhaber N HAE patient self-sampling for biomarker establishment. Orphanet J Rare Dis. (2021) 16:399. 10.1186/s13023-021-02021-x 34583739 PMC8478266

[48] Isono M Kokado M Kato K. Why does it take so long for rare disease patients to get an accurate diagnosis?—a qualitative investigation of patient experiences of hereditary angioedema. PLoS One. (2022) 17:e0265847. 10.1371/journal.pone.0265847 35303740 PMC8932585

[49] Zanichelli A Magerl M Longhurst H Fabien V Maurer M. Hereditary angioedema with C1 inhibitor deficiency: delay in diagnosis in Europe. Allergy Asthma Clin Immunol. (2013) 9(1):29 10.1186/1710-1492-9-29 23937903 PMC3751114

[50] Savarese L Bova M De Falco C Guarino MD De Luca Picione R Petraroli A Emotional processes and stress in children affected by hereditary angioedema with C1-inhibitor deficiency: a multicenter, prospective study. Orphanet J Rare Dis. (2018) 13:115. 10.1186/s13023-018-0871-x 30005674 PMC6043996

[51] Savarese L Freda MF De Luca Picione R Dolce P De Falco R Alessio M The experience of living with a chronic disease in pediatrics from the mothers’ narrative: the clinical interview on parental sense of grip on the disease. Health Psychol Open. (2020):1–13. 10.1177/2055102920971496 PMC7727074 33343914

[52] Bork K Anderson JT Caballero T Craig T Johnston DT Li HH Assessment and management of disease burden and quality of life in patients with hereditary angioedema: a consensus report. Allergy Asthma Clin Immunol. (2021) 17:40. 10.1186/s13223-021-00537-2 33875020 PMC8056543

[53] Farkas H. Pediatric hereditary angioedema due to C1-inhibitor deficiency. Allergy Asthma Clin Immunol*.* (2010) 6:18. 10.1186/1710-1492-6-18 20667121 PMC2920237

[54] Nanda MK Elenburg S Bernstein J Assa’ad AH. Clinical features of pediatric angioerdema. J Allergy Clin Immunol Pract*.* (2015) 3:392–5. 10.1016/j.jaip.2014.11.012 25609346 PMC8207479

[55] Christiansen SC Davis DK Castaldo AJ Zuraw BL. Pediatric hereditary angioedema: onset, diagnostic delay, and disease severity. Clin Pediatr. (2016) 55:935–42. 10.1177/0009922815616886 26581355

[56] Karadža-Lapić L Barešić M Vrsalović R Ivković-Jureković I Sršen S Prkačin I Hereditary angioedema due to C1-inhibitor deficiency in pediatric patients in Croatia—first national study, diagnostic and prophylactic challenges. Acta Clin Croat*.* (2019) 58: 139–46. 10.20471/acc.2019.58.01.18 31363336 PMC6629194

[57] Cancian M Perego F Senter R Arcoleo F De Pasquale T Zoli A Pediatric angioedema: essential features and preliminary results from the hereditary angioedema global registry in Italy. Pediatr Allergy Immunol. (2020) 31(Suppl 24):22–4. 10.1111/pai.13170 32017221

[58] Andrási N Balla Z Szilági A Csuka D Varga L Farkas H. Diagnosing pediatric patients with hereditary C1-inhibitor deficiency—experience from the Hungarian center of reference and excellence. Front Allergy*.* (2022) 3:860355. 10.3389/falgy.2022.860355 35769571 PMC9234934

[59] Patton GC Viner R. Pubertal transitions in health. Lancet (2007) 369(9567):1130–9. 10.1016/S0140-6736(07)60366-3 17398312

[60] Zanichelli A Magerl M Longhurst HJ Aberer W Caballero T Bouillet L Improvement in diagnostic delays over time in patients with hereditary angioedema: findings from the Icatibant Outcome Survey. Clin Transl Allergy. (2018) 8:42. 10.1186/s13601-018-0229-4 30338053 PMC6182796

[61] Walker IV Smith CR Davies JH Inskip HM Baird J. Methods for determining pubertal status in research studies: literature review and opinions of experts and adolescents. J Dev Orig Health Dis. (2020) 11(2):168–87. 10.1017/S2040174419000254 31204632

[62] Kim T Yun JW Son M Kim CB Choe SA. Age at menarche of adolescent girls and the neighbourhood socioeconomic status of their school area. Eur J Contracept Reprod Health Care. (2022):1–7. 10.1080/13625187.2022.2104834 36053277

[63] Suutela M Miettinen PJ Kosola S Rahkonen O Varimo T Tarkkanen A Timing of puberty and school performance: a population-based study. Front Endocrinol (Lausanne)*.* (2022) 13:936005. 10.3389/fendo.2022.936005 35992102 PMC9388756

[64] Aabom A Andersen KE Fagerberg C Fisker N Jakobsen MA Bygum A. Clinical characteristics and real-life diagnostic approaches in all Danish children with hereditary angioedema. Orphanet J Rare Dis. (2017) 12(1):55. 10.1186/s13023-017-0604-6.63 28302171 PMC5356294

[65] Bork K Meng G Staubach P Hardt J. Hereditary angioedema: new findings concerning symptoms, affected organs, and course. Am J Med. (2006) 119(3):267–74. 10.1016/j.amjmed.2005.09.064.b 16490473

[66] Piotrowicz-Wójcik K Bulanda M Juchacz A Jamróz-Brzeska J Gocki J Kuziemski K Clinical characteristics and management of angioedema attacks in Polish adult patients with hereditary angioedema due to C1-inhibitor deficiency. J Clin Med. (2021) 10(23):5609. 10.3390/jcm10235609 34884311 PMC8658320

[67] Bouillet L Gompel A. Hereditary angioedema in women: specific challenges. Immunol Allergy Clin North Am*.* (2013) 33(4):505–11. 10.1016/j.iac.2013.07.006 24176215

[68] Caballero T Farkas H Bouillet L Bowen T Gompel A Fagerberg C International consensus and practical guidelines on the gynecologic and obstetric management of female patients with hereditary angioedema caused by C1 inhibitor deficiency. J Allergy Clin Immunol*.* (2012) 129(2):308–20. 10.1016/j.jaci.2011.11.025 22197274

[69] Agostoni A Cicardi M. Hereditary and acquired C1-inhibitor deficiency: biological and clinical characteristics in 235 patients. Medicine (Baltimore)*.* (1992) 71(4):206–15. 10.1097/00005792-199207000-00003 1518394

[70] Triggianese P Senter R Petraroli A Zoli A Lo Pizzo M Bignardi D Pregnancy in women with hereditary angioedema due to C1-inhibitor deficiency: results from the ITACA cohort study on outcome of mothers and children with *in utero* exposure to plasma-derived C1-inhibitor. Front Med (Lausanne)*.* (2022) 9:930403. 10.3389/fmed.2022.930403 36186797 PMC9515414

[71] Kulkarni M Travers JB Rohan C. High estrogen states in hereditary angioedema: a spectrum. Clin Rev Allergy Immunol. (2021) 60(3):396–403. 10.1007/s12016-021-08863-4 34075568

[72] Gompel A Fain O Boccon-Gibod I Gobert D Bouillet L. Exogenous hormones and hereditary angioedema. Int Immunopharmacol. (2020) 78:106080. 10.1016/j.intimp.2019.106080 31855692

[73] Mawhinney M Mariotti A. Physiology, pathology and pharmacology of the male reproductive system. Periodontol 2000. (2013) 61(1):232–51. 10.1111/j.1600-0757.2011.00408.x 23240952

