# Supplementary material for: The impact of puberty on the onset, frequency, location, and severity of attacks in hereditary angioedema due to C1-inhibitor deficiency: A survey from the Italian Network for Hereditary and Acquired Angioedema (ITACA)
Source: Front Pediatr. 2023 Apr 18;11:1141073. doi: 10.3389/fped.2023.1141073 (PMC10152551; doi:10.3389/fped.2023.1141073)
Supplement: Supplementary file 1 [file Table1.docx]

Supplementary Material

**The impact of puberty on onset, frequency, location and severity of attacks in Hereditary Angioedema due to C1-inhibitor deficiency: a survey from the Italian Network for Hereditary and Acquired Angioedema (ITACA)**

Mauro Cancian ^1^*, Paola Triggianese ^2^, Stella Modica^2^, Francesco Arcoleo ^3^, Donatella Bignardi ^4^, Luisa Brussino ^5^, Caterina Colangelo ^6^, Ester Di Agosta ^7^, Davide Firinu ^8^, Maria Domenica Guarino ^9^, Francesco Giardino ^10^, Marica Giliberti ^11^, Vincenzo Montinaro ^12^, Riccardo Senter ^1^

^1^ University Hospital of Padua, Padua, Italy, ^2^ Tor Vergata University Hospital, Rome, Italy, ^3^ Azienda Ospedaliera Ospedali Riuniti Villa Sofia-Cervello, Palermo, Italy, ^4^ IRCCS Ospedale Policlinico San Martino, Genova, Italy, ^5^ University Hospital Ordine Mauriziano di Torino, Turin, Italy, ^6^ Azienda Sanitaria Locale di Pescara, Pescara, Italy, ^7^Careggi University Hospital, Florence, Italy, ^8^ University Hospital of Cagliari, Cagliari, Italy, ^9^ Ospedale di Civitanova Marche, Civitanova Marche, Italy, ^10^ Policlinico Universitario di Catania, Catania, Italy, ^11^ Azienda Ospedaliero-Universitaria Policlinico di Bari, Bari, Italy, ^12^ Ospedale Regionale Generale F Miulli, Acquaviva delle Fonti, Italy

Correspondence: Mauro Cancian [mcancian@unipd.it](mailto:mcancian@unipd.it)

| Gender | Male □ Female □ |  |
| --- | --- | --- |
| Birth date | (dd/mmm/yyyy) |  |
| Current age | years |  |
| Age at diagnosis | years |  |
| Age of symptom’s onset | years |  |
| Age of puberty | years |  |
| Affected parent | No□ mother □ father □ |  |
| Siblings | No□ Yes (how many: _____, of whom affected:______) |  |
| Monthly mean of attacks the year before puberty |  |  |
| Monthly mean of attacks in the three years before puberty |  |  |
| Treatments in the emergency room before puberty | ______ |  |
| Main attack location before puberty | cutaneous □ abdominal □ upper airways □ other □ |  |
| First on demand treatment used | Berinert □ Firazyr □ Tranex □ Cinryze □ None □ |  |
| Main on demand treatment before puberty | Berinert □ Firazyr □ Tranex □ Cinryze □ None □ |  |
| Prophylaxis used before puberty | Tranex □ Danazol □ Cinryze □ None □ |  |
| Monthly mean of attacks the year after puberty |  |  |
| Monthly mean of attacks in the three years after puberty |  |  |
| Treatments in the emergency room after puberty |  |  |
| Main attack location after puberty | cutaneous □ abdominal □  upper airways □ other □ |  |
| Main on demand treatment before puberty | Berinert □ Firazyr □ Tranex □ Cinryze □  None □ | |
| Prophylaxis used before puberty | Tranex □ Danazol □ Cinryze □ None □ |  |
| Current treatments non-HAE related |  |  |
| Subjective feelings of symptoms after puberty | more intense □ more frequent □ improved □ no change □ |  |
| If worsened (symptoms more intense/more frequently) | permanently □ transiently □ |  |

**Supplementary Table 1:** Questionnaire administered to the patients

| Univariate logistic regression: |
| --- |
| Dependent variables: worsening of symptom 1 year after puberty, worsening of symptom 3 years after puberty |
| Independent variable: gender |
|  |
| Multivariate logistic regression: |
| Dependent variable: worsening of symptom 1 year after puberty |
| Independent variables: gender, age of onset of puberty |
| Dependent variable: worsening of symptom 3 years after puberty |
| Independent variables: gender, age of onset of puberty |

**Supplementary Table 2:** Regression analyses
